# Preparation of Double-Layer Composite Coffee Filtration Nonwovens

**DOI:** 10.3390/polym16162275

**Published:** 2024-08-10

**Authors:** Lihuan Zhao, Yujie Yang, Yuwen Wang, Ziyan Yan, Rong Zhang

**Affiliations:** 1 School of Textile Science and Engineering, Tiangong University, Tianjin 300387, China; yangdashuai2020@163.com (Y.Y.); yanzy9528@163.com (Z.Y.); zr07032024@163.com (R.Z.); 2 Key Laboratory of Advanced Textile Composites, Ministry of Education, Tiangong University, Tianjin 300387, China; 3 Chybond Materials Co., Ltd., Tianjin 300380, China; jacky.wang@zoefychina.com

**Keywords:** coffee filtration nonwovens, double-layer composite nonwovens, the filtration of coffee, coffee filtration rules

## Abstract

The coffee industry is developing rapidly in the world, and the use of coffee filtration nonwovens (CFNs) is becoming more and more extensive; however, there is a lack of standards and research for its production and trade, and the quality of related products on the market is uneven at present. Here, eight double-layer composite coffee filtration nonwovens (D-LCCFNs) were prepared by using 5 g/m^2^ and 10 g/m^2^ polypropylene (PP) melt-blown nonwovens (MNs), 20 g/m^2^ PP spunbonded nonwovens and 20 g/m^2^ viscose/ES fiber chemically bonded nonwovens, and the physical properties, morphology and the filtration effect of coffee and purified water for the prepared samples were tested. It was found that the surface density of the microfiber layer (MNs) in the D-LCCFNs was negatively correlated with the coffee filtration rate; when the microfiber layer in the D-LCCFNs was in direct contact with the coffee, the liquid started to drip later, and the filtration rate of the coffee was slower; the filtration rate of the samples with the viscose/ES chemically bonded nonwovens was very fast. However, the samples without viscose/ES fibers basically did not filter pure water much, but they could filter out the coffee liquid normally, and the samples’ hydrophilicity increased significantly after filtering coffee.

## 1. Introduction

The history of coffee drinking began thousands of years ago in the African plateau [1]; coffee is native to the Ethiopian and Congolese regions of Africa [2]. Currently, more than 70 countries have developed coffee cultivation, and the most common cultivars are Arabica and Robusta [3]. Among them, Arabica coffee, also known as small-grain coffee, accounts for 70% of the coffee market [4,5]. Arabica coffee has an excellent flavor and unique aroma and is the only one of these native species that can be consumed directly and separately. The origin of the coffee, the degree of roasting and the brewing method all affect the flavor of the coffee [6,7,8]. Coffee cultivation in China is mainly concentrated in Yunnan, Hainan and Sichuan, and the low latitude and high altitude conditions in Yunnan give the Yunnan coffee beans a sweet caramel aroma and a slightly acidic character, which makes them universally praised [9]. In recent years, China’s coffee industry has been developing at a relatively fast pace, and studies have shown that China’s coffee consumption market has potential coffee consumers amounting to 200 million to 250 million, with huge room for development. The degree of coffee roasting, brewing method and filtration method will all have a certain impact on the flavor of coffee. In the beginning, people used flannel fabrics to filter coffee, as the flannel surface is soft and delicate, with no hair, pilling, shrinkage and other phenomena. Its pore size is larger, can be more filtered out of coffee oil, and of course, inevitably, flannel cannot effectively intercept the fine particles in the coffee. Later, coffee filter paper was widely adopted and greatly respected. The filter paper draws on the characteristics of the flannel filter cloth with a fine cloth surface and a thick suede surface. The two different densities of the filter paper being pressed together reduces the phenomenon that the single density filter paper is easy to make the coffee particles be retained. Until now, coffee filter paper is still retained in one side of the market. 

Coffee filtration/extraction methods are constantly iterating and innovating to meet consumers’ demands for a better and more diverse coffee experience. With the continuous updating of coffee consumption forms, in addition to freshly ground coffee, capsule coffee, cold brew coffee and hanging ear coffee have been invented that are simpler and more convenient and can retain the coffee flavor to a large extent. These new forms of coffee mostly use nonwovens for coffee filtration, probably because nonwovens have the advantages of high strength, good filtration performance and strong designability. Currently, nonwovens are widely used in various liquid filtration fields [10,11,12]. For capsule coffee, cold brew and hanging ear coffee, the type or specification of the non-woven fabric used to achieve filtration is different because of the different coffee flavors they correspond to. Hanging ear coffee is a ready-to-brew type of freshly ground coffee, which is brewed by drip filtration. In order to achieve the brewing effect of hanging ear coffee, double-layer composite coffee filtration nonwovens (D-LCCFNs) containing a layer of microfibers are generally used for packaging and filtration. According to market research, most manufacturers choose to use polypropylene (PP) to prepare D-LCCFNs for hanging ear coffee filtration, with specifications typically around 30 g/m^2^, which is made of a layer of meltblown nonwovens (MNs) and a layer of ordinary spunbond nonwovens thermally bonded. MNs are considered to be the most promising filtration material due to their low air resistance and high filtration performance [13,14,15]. Its high surface area and excellent barrier properties are also considered to be useful in improving filtration efficiency [16,17].

As an emerging coffee filter material, the research on coffee filtration nonwovens (CFNs) is still in its infancy at home and abroad. At present, there are no international standards, national standards or industry standards for the preparation of CFNs, and there are few reports on the effect of CFNs on coffee filtration, which is very unfavorable to the development and application of CFNs. Therefore, based on the results of market research, we prepare D-LCCFNs for hanging ear coffee and explore the performance and coffee filtration rules of D-LCCFNs to provide a reference for the further development of D-LCCFNs.

## 2. Materials and Methods

### 2.1. Materials

Both 5 g/m^2^ and 10 g/m^2^ PP MNs were purchased from Tianjin TEDA Clean Materials Co., Ltd., (Tianjin, China). According to the results of the preliminary pre-experiment, when 15 g/m^2^ PP MN was thermally bonded with 10~25 g/m^2^ PP spunbond nonwoven, the composite nonwoven was difficult to filter out the coffee liquid, which might be due to the fact that the grammage of the MN was too large, the fiber layer was dense and the fiber gap was too small, which resulted in the coffee liquid being unable to pass through it. However, the preparation of PP MNs with a weight of less than 5 g/m^2^ is difficult, resulting in extremely high costs. Therefore, considering the product cost and production difficulty as well as the filtration effect on the coffee liquid, the PP MNs of 5 g/m^2^ and 10 g/m^2^ were selected in this experiment.

Both 20 g/m^2^ of PP spunbond nonwovens and 20 g/m^2^ of chemically bonded nonwovens with viscose/ES fibers (blend ratio 3:7) were obtained from Tianjin Qibang New Materials Co., Ltd., (Tianjin, China). The grammage of the samples was chosen with reference to the results of market research on D-LCCFNs.

The coffee powder, selected from a brand with over 100,000 sales on a Chinese platform, was a medium-roasted small-grain coffee powder from Yunnan province, China, with a neutral flavor and taste, which was suitable for hanging ear coffee.

For easy understanding, the experimental samples were named as follows: 5 g/m^2^ PP MN named “A5”, 10 g/m^2^ PP MN named “A10”, 20 g/m^2^ PP spunbonded nonwoven named “B” and 20 g/m^2^ viscose/ES chemically bonded nonwoven named “C”. 

Since the water contact angle test, coffee filtration test and pure water filtration test were involved in this study, the following nomenclature was carried out for ease of understanding: two layers of nonwovens, expressed as “upper/lower”, with the upper layer for the test layer, which was used with coffee/pure water as a direct contact layer or tested layer; a combination such as A5/B was obtained by compounding A5 and B, with the A5 for the upper layer (coffee/pure water direct contact layer or tested layer); B/A5 was still obtained by compounding A5 and B but with B as the upper layer (coffee/pure water direct contact layer or tested layer). 

### 2.2. Preparation of Samples

The SIMATIC HMI composite equipment was used to hot-roll B and C with A5 and A10, respectively, and the process parameters used in compounding were upper and lower roll temperatures of 130 °C, pressure of 55 N and speed of 15 m/min. Eight samples were obtained from A5/B, B/A5, A10/B, B/A10, A5/C, C/A5, A10/C and C/A10. 

### 2.3. Scanning Electron Microscopy (SEM), Contact Angle Test and Fourier Transform Infrared Spectroscopy

Using the desktop scanning electron microscope (Phenom XL), each of the eight samples was attached to the sample stage with conductive adhesive, sprayed with gold and loaded into the instrument compartment to begin observation photography.

The hydrophilicity of the sample surface was characterized using a video optical contact angle measuring instrument (OCA15pro), and the contact angle measurement of the sample surface was performed by using the SCA20 contact angle measurement syetem came with the instrument. 

Fourier transform infrared (FTIR) spectroscopy (Nicolet iS50) was utilized to analyze the functional groups of coffee powder and D-LCCFNs before and after filtering coffee, with Nicolet iS50 capable of testing infrared spectra in the mid-infrared range (4000–500 cm^−1^).

### 2.4. Coffee/Pure Water Filtration Test

**Figure 1:**
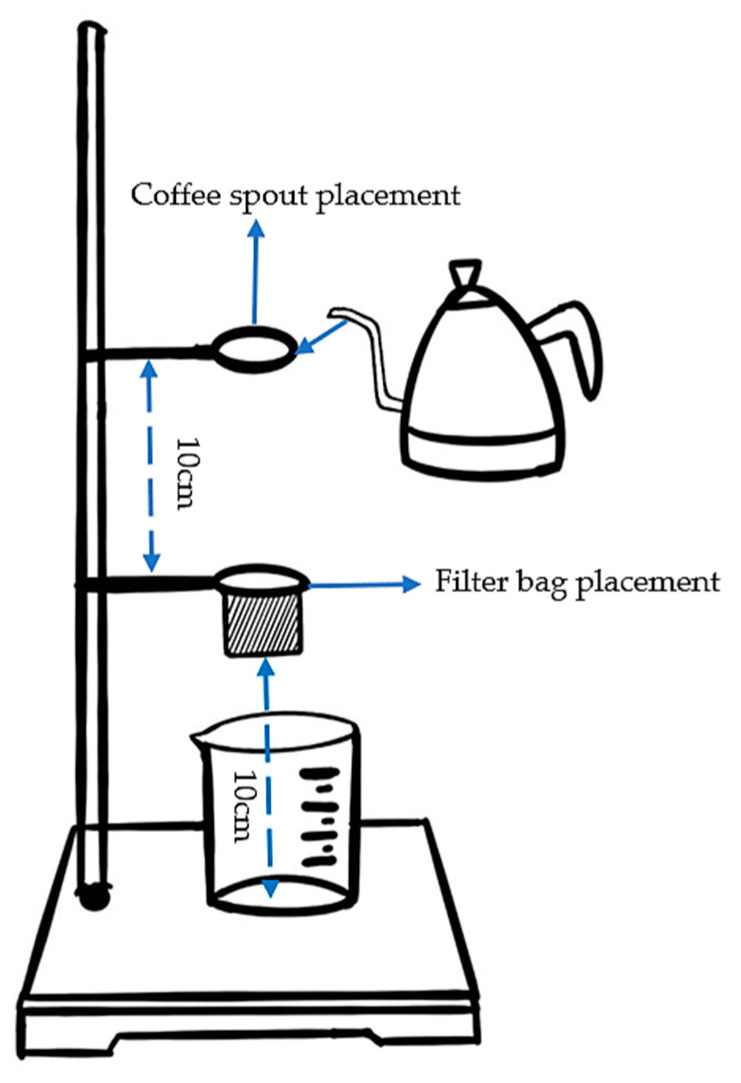
Simple diagram of coffee brewing.

The prepared D-LCCFNs were used as samples for coffee brewing, and the specific experimental procedure for each sample was as follows: (1) A piece of 15 cm × 15 cm specimen was randomly cut from the sample and folded in half, and a straight plate clamp preheated to 160 °C was used to press the folded specimen at a distance of 3 cm on both sides of the specimen to make it bonded, and then a stapler was used to fix the specimen on both sides of the specimen again, and the appearance of the processed specimen was similar to that of the hanging ear coffee filter bag. (2) The beaker was placed on the pedestal and the prepared specimen was fixed on the experimental iron stand with clamps on both sides, respectively, so that the specimen was an open bag and distance from the bottom of the specimen to the bottom of the beaker was about 10 cm; we used an electronic balance to weigh 1 portion of 5 g weight of coffee powder and poured it into the specimen bag. (3) An iron ring was fixed about 10 cm above the mouth of the specimen so that the spout of the coffee pot was always kept at the height of the iron ring to control the brewing height. (4) The coffee was filtered using 80 mL of hot water at 90 °C; during the brewing process, the coffee pot was tilted at an angle of 45° to maintain an even brewing rate. (5) The time when the coffee liquid started to drip during the filtration process was recorded, as well as the total amount of coffee liquid filtered out from that moment at 20 s intervals, and the brewed coffee lasted for 3 min. A simple schematic diagram for brewing coffee is shown in Figure 1.

In the previous study, when the pre-experiment of coffee filtration was carried out with the purchased D-LCCFNs, it was found that there was a large difference in the effect of the nonwoven fabric in filtering coffee and filtering water. Therefore, to explore the difference between the D-LCCFNs in filtering coffee and filtering purified water, as well as the mechanism of the nonwoven fabric filtering coffee, we also designed an experiment to filter pure water with D-LCCFNs.

The prepared D-LCCFNs were used as samples for pure water filtration experiments. No coffee powder was added in this experiment, the preparation method of the hanging ear coffee filter bag sample was the same as the preparation method of the sample in the above-mentioned coffee filtration experiment and the experimental process of filtering water was also the same as the experimental process of filtering coffee. 

## 3. Results and Discussion

### 3.1. SEM Results and Analysis

**Figure 2:**
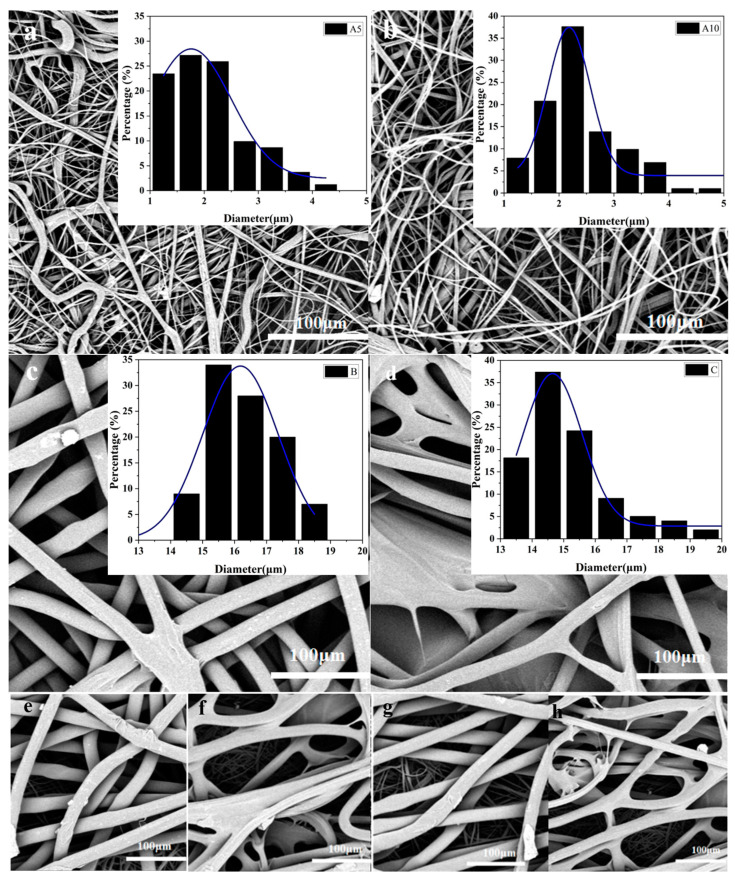
Electron microscopy and fiber distribution of the samples (**a**) A5, (**b**) A10, (**c**) B, (**d**) C, (**e**) B/A5, (**f**) B/A10, (**g**) C/A5 and (**h**) C/A10.

The results of the morphology and fiber diameter distribution of each sample are shown in Figure 2. 

Figure 2a,b show the PP MNs. It could be seen that the fiber arrangement was more chaotic, and there was a link between the fibers entangled. The fiber fineness was not uniform, and the fiber fineness was 1 μm~5 μm, which belonged to the ultrafine fibers, of which the fibers with a diameter of about 2 μm account for the largest percentage. Figure 2c shows PP spunbond nonwovens. The flattened part of the figure was the rolling point, and its fiber diameter was distributed within 14 μm and 19 μm, and about 70% of the fibers were around 16 μm. Figure 2d represents the viscose/ES chemically bonded nonwovens. It could be clearly seen that the fibers had gelatinous substances, which might be caused by the gluing process in their preparation, and the fiber diameter was 13 μm~20 μm, but the fibers less than 16 μm accounted for a relatively large proportion, about 80%. Figure 2f–h show the composite nonwovens, from which it was obvious that the composite nonwovens had a bilayer structure, with the upper layer being the nonwovens with thicker fibers (B or C) and the lower layer being the nonwovens with finer fibers (A5 or A10).

### 3.2. Contact Angle Test Results and Analysis

**Figure 3:**
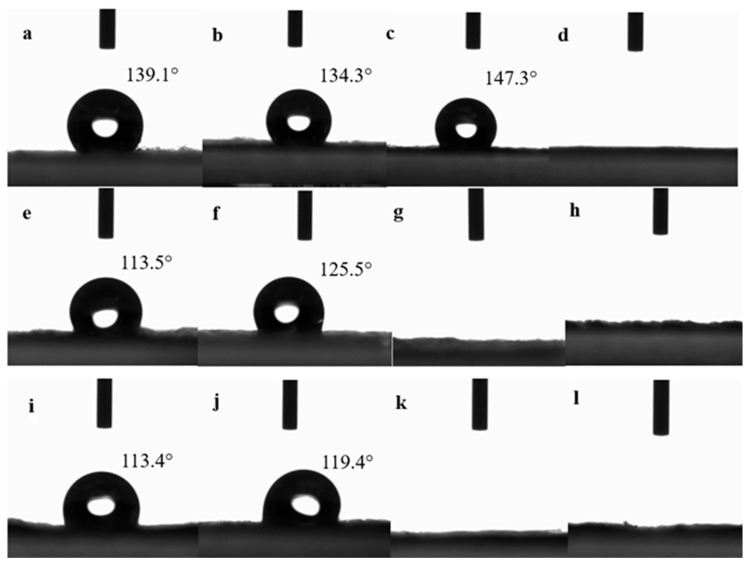
Contact angle test results of the samples (**a**) A5, (**b**) A10, (**c**) B, (**d**) C, (**e**) A5/B, (**f**) B/A5, (**g**) A5/C, (**h**) C/A5, (**i**) A10/B, (**j**) B/A10, (**k**) A10/C and (**l**) C/A10.

The contact angle test results of the samples are shown in Figure 3:

As shown in Figure 3, the samples containing C were very hydrophilic no matter which side was in contact with the water droplet, while the samples without C were very hydrophobic. The hydrophilicity of A5, A10 and B were very poor, and their contact angles were 139.1°, 134.3° and 147.3°, respectively. When they were laminated with each other (such as A5/B, B/A5, A10/B and B/A10), however, their contact angles were significantly reduced, which might be due to the roughness of the surface of the fabrics that also affected the transfer of liquid water. After the hot roller lamination, the fabric surface became smooth and the roughness decreased, the contact area of water droplets with the fabric surface became larger and therefore, the contact angle decreased. 

### 3.3. Coffee Filtration Experiment Results and Analysis

The following are the experimental results and analysis of samples of filtering coffee. In the legends of the figures below, the numbers in parentheses are the times when the samples start to filter out liquid, in seconds (s):

- Effect of hydrophobic and hydrophilic nonwovens compounded with A5 on coffee filtration effect.

**Figure 4:**
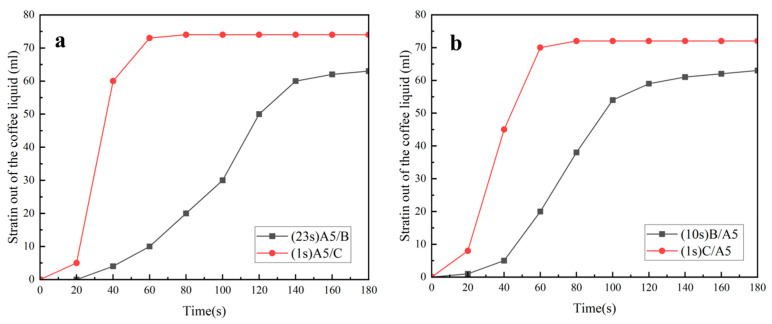
Experiment on the coffee filtration effect of D-LCCFNs on coffee filtration (**a**) comparison between A5/B and A5/C, (**b**) comparison between of B/A5 and C/A5.

As shown in Figure 4a, when the coffee was filtered, A5/B started to drip at 23 s, the rate of dripping filtration first increased and then decreased, the filtration was completed at 180 s and the filtered coffee liquid was about 65 mL. A5/C started to drip at the first second, the speed of the dripping filtration was very fast, 60 mL of the coffee liquid was filtered out at 40 s and about 74 mL of the coffee liquid was filtered out at about 60 s. 

As can be seen from Figure 4b, when filtering coffee, B/A5 began to drip at 10 s, the rate of drip filtering first increased and then decreased, the filtration was completed at 180 s and the filtered coffee liquid was about 65 mL. C/A5 also started to drip at the first second, the rate of drip filtering was very fast, 70 mL of coffee liquid had been filtered out at 60 s and the filtering of the coffee liquid was completed at this time. At 80 s, the filtration was completely finished, and 74 mL of coffee liquid was filtered out. 

In summary, regardless of whether the A5 MN was the upper or lower layer, the composite nonwoven with hydrophilic nonwoven (C) had a faster filtration rate when filtering coffee, the final coffee liquid filtered out of the D-LCCFNs with C was about 74 mL and the D-LCCFNs without C filtered out about 65 mL of coffee liquid.

- Effect of hydrophobic and hydrophilic nonwovens compounded with A10 on coffee filtration effect.

**Figure 5:**
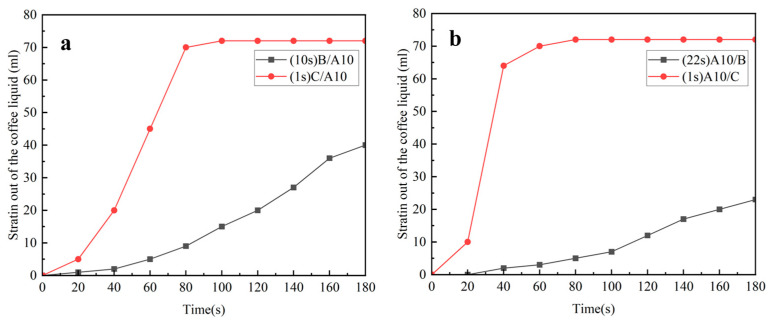
Experiment on the coffee filtration effect of D-LCCFNs on coffee filtration (**a**) comparison between B/A10 and C/A10, (**b**) comparison between of A10/B and A10/C.

Figure 5a shows the coffee filtration results of B/A10 and C/A10. When filtering coffee, B/A10 started to drip at 10 s and filtered about 40 mL of coffee at 180 s, which was not completed. C/A10 started to drip coffee liquid at the first second and dripped very fast, which ended the filtration at 80 s and filtered about 74 mL of coffee liquid. 

Figure 5b shows the coffee filtration results of A10/B and A10/C. A10/B began to drip coffee liquid at 22 s, the drip rate increased slowly and less than 30 mL of coffee liquid was filtered out at 180 s. A10/C began to drip at the first second, the drip speed was very fast, 65 mL of coffee liquid had been filtered out at 40 s and then the drip speed was slowed down. The filtration was completed at about 80 s, and the filtered coffee liquid was about 72 mL. 

In summary, whether the A10 MN was in the upper or lower position, its composite nonwoven with hydrophilic nonwoven (C) filtered coffee faster, and the final amount of coffee liquid filtered out of the D-LCCFN with C was more than 34 mL higher than that of the D-LCCFN with hydrophobic nonwoven (B).

- The impact on the coffee filtration effect when MNs of different weights (A5, A10) are compounded with hydrophobic and hydrophilic nonwovens and with MNs are the upper layer.

**Figure 6:**
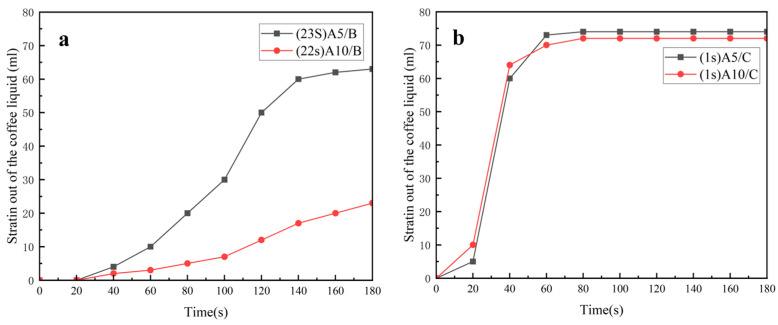
Experiment on the coffee filtration effect of D-LCCFNs on coffee filtration (**a**) comparison between A5/B and A10/B, (**b**) comparison between A5/C and A10/C.

Figure 6a shows that when filtering coffee, A5/B started to drip in 23 s, the drip filtration rate first increased and then decreased, the filtration was completed in 180 s and the filtered coffee liquid was about 65 mL. When filtering coffee, A10/B began to drip in 22 s, the drip rate increased slowly and less than 30 mL of coffee liquid was filtered out in 180 s. 

As shown in Figure 6b, A5/C started to drip at the first second at a very fast speed, 60 mL of coffee was filtered out in 40 s and the filtration was completed at about 60 s, with about 74 mL of coffee filtered out. While the A10/C nonwoven also started to drip coffee liquid at the first second and the drip filtration speed was very fast, 65 mL of coffee was filtered out in 40 s, and the filtration speed slowed down at about 80 s, with about 72 mL of coffee liquid being filtered out. Afterward, the drip filtration slowed down and the filtering was completed in about 80 s, resulting in about 72 mL of coffee liquid. 

From the above, it can be concluded that when filtering coffee with MN as the upper layer, regardless of whether it was compounded with hydrophobic or hydrophilic nonwoven, the composite nonwoven with A5 was filtered faster than the composite nonwoven with A10, and finally, the amount of final coffee liquid filtered out of the composite nonwoven with A5 was higher than that of the composite nonwoven with A10.

- The impact on the coffee filtration effect when MNs of different weights (A5, A10) are compounded with hydrophobic and hydrophilic nonwovens and with MNs are the lower layer.

**Figure 7:**
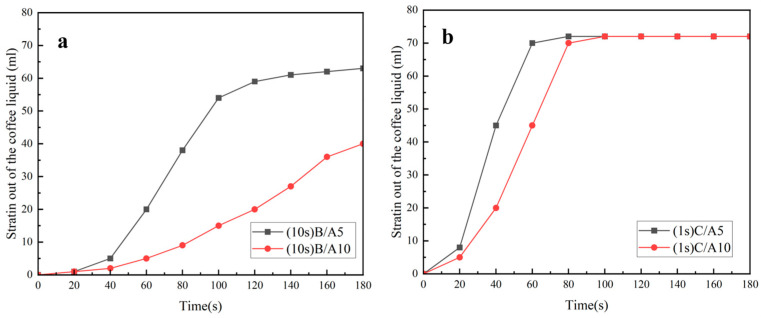
Experiment on the coffee filtration effect of D-LCCFNs on coffee filtration (**a**) comparison between B/A5 and B/A10, (**b**) comparison between C/A5 and C/A10.

As shown in Figure 7a, when filtering coffee, both B/A5 and B/A10 started to drip in 10 s, and the drip filtration speed showed a trend of increasing and then decreasing, but the filtration speed of the former was faster than that of the latter, and the former had completed the filtration in 180 s and filtered out about 64 mL of coffee liquid, while the latter did not complete filtration in 180 s and filtered out about 40 mL of coffee liquid at this time. 

As can be seen from Figure 7b, both C/A5 and C/A10 started filtering at the first second, and both drip filtration speeds were very fast, but the filtration speed of the former was faster than that of the latter, and both had basically ended filtration in 80 s. The final filtered coffee liquid was about 74 mL in both cases. 

By comparing Figure 7a,b, it can be seen that when the MNs were the lower layer, whether they were laminated with hydrophobic or hydrophilic nonwovens, the compounded nonwovens with A5 filtered out the coffee significantly faster than the compounded nonwovens with A10. When the MNs were compounded with B, and the grammage of MNs was increased from 5 g/m^2^ to 10 g/m^2^, the amount of coffee liquid filtered out decreased significantly. In 180 s, the B/A5 filtered out 64 mL of coffee liquid, which was already filtered out, while the B/A10 filtered out less than 40 mL of coffee liquid. C/A5 and C/A10 both filtered out around 80 s, and the final amount of coffee filtered out was almost the same.

- Effect on the coffee filtration effect when A5 was compounded with B and C, and when A5 was the upper or lower layer, respectively.

**Figure 8:**
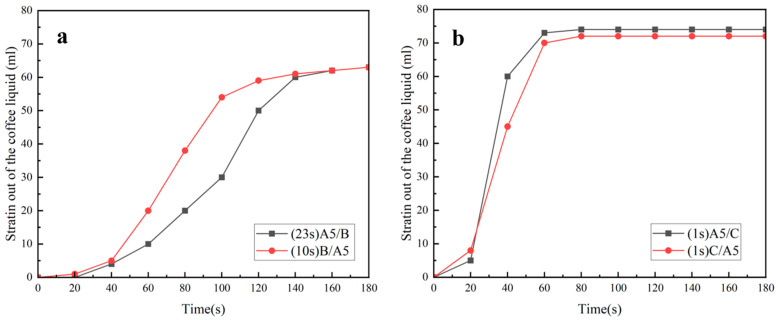
Experiment on the coffee filtration effect of D-LCCFNs (**a**) comparison between B/A5 and A5/B, (**b**) comparison between of C/A5 and A5/C.

Figure 8a shows that A5/B and B/A5 started drip filtration in 23 s and 10 s, respectively. It can be seen from the figure that the filtration speed of the former was slower than that of the latter, but the end of the filtration time and the content of the final filtered coffee liquid were almost the same, and the final filtered coffee liquid was 64 mL. 

As shown in Figure 8b, the drip filtration time of A5/C and C/A5 was the same, and the drip filtration started at the first second. From the figure, it can be seen that the filtration speed of the former was slightly faster than that of the latter, but the final end of the filtration time and the content of the final filtered coffee liquid were similar, and the final filtered coffee liquid of A5/C and C/A5 were 74 mL and 72 mL, respectively. 

In summary, when A5 was compounded with B and C, whether A5 was the upper or lower layer would affect the rate of coffee filtration, and the situation was just the opposite for B and C. When it was compounded with B and was the lower layer, its coffee filtration rate was faster. But when it was compounded with C, its coffee filtration rate was faster when it was the upper layer.

- The effect on the coffee filtration when A10 was compounded with B and C, and when A10 was the upper or lower layer, respectively.

**Figure 9:**
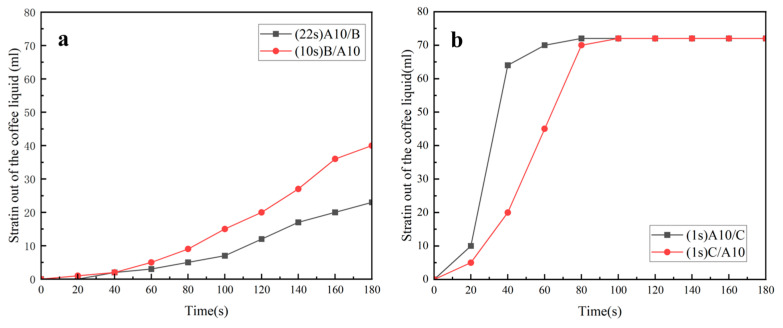
Experiment on the coffee filtration effect of D-LCCFNs (**a**) comparison between B/A10 and A10/B, (**b**) comparison between of C/A10 and A10/C.

As shown in Figure 9a, A10/B began to drip at 22 s, while B/A10 began to drip at 10 s. The filtration speed of both was gradually increasing, the drip rate of the former was slower than that of the latter, and the filtration of both was not completed at 180 s, but the latter filtered out more coffee liquid at this time.

Figure 9b shows that A10/C and C/A10 began to drip at 1 s, the former filtration speed was faster than the latter, but the end of the filtration time and the final filtration of the content of the coffee solution were almost the same; both basically completed filtration at 80 s, and the final filtration volumes of the coffee liquid were all about 74 mL.

In summary, the composite nonwovens of A10 with B and C had the same filtration law for coffee liquid as the composite nonwovens of A5 with B and C.

Summarizing the above experimental results and analysis, the following six conclusions can be drawn:

(1) Regardless of whether the MNs were the upper or lower layer, their composite nonwovens with hydrophilic nonwoven (C) had a faster coffee drip rate when filtering coffee, and the amount of coffee liquid filtered out of the composites containing C was larger than that of the nonwovens without C.
(2) This may be due to the hydrophilicity of C being very good, and the hydrophilicity of the composite nonwovens with C was also good. The upper and lower layers of the contact angle were basically 0°, so when the coffee was filtered, the liquid just contacted the composite nonwovens and immediately penetrated through, so that the composite nonwovens containing C finally filters out a larger amount of coffee liquid than the composite nonwovens without C.
(3) Whether the MNs were the upper or lower layer, and whether they were compounded with B or C, the composite nonwovens with A5 filter coffee faster than the composite nonwovens with A10. This might be due to the fact that A5 had a lower surface density than A10, its fiber network arrangement was relatively sparse, and its porosity and average pore size were larger than that of A10, which made the nonwovens with A5 filter coffee faster than the nonwovens of with A10.
(4) When compounded with B, whether it was A5 or A10, when filtering coffee, the coffee filtration rate was smaller when A5 and A10 were the upper layers, and the drip filtration started later when A5 and A10 were the upper layers than when the A5 and A10 were the lower layers. This might be due to the fact that the fiber mesh of the MN layer was more densely arranged. When it was the upper layer, the coffee filtering conditions were more stringent and fine particles were directly blocked.
(5) In addition, this might also be related to the differential capillary effect. The composites of A5 and the composites of A10 were both double-layer structures, at which time the aperture of the fiber network of the B layer and the C layer was larger, and the pressure of the capillary tube was attached to a smaller one. The junction between the two layers of the composite nonwovens would form a pressure difference, resulting in the liquid being transferred from the B and C layers to the MNs layer, and when the B and C layers were the upper layer, this differential capillary effect sped up the rate of the coffee filtration, whereas this effect could not be produced when A5 and A10 were the upper layers.
(6) When compounded with C, both A5 and A10 MNs filtered coffee faster when the MNs were the upper layer, but there was no change for the start of the drip filtration time. In general, it could be seen that the start time of the drip filtration of the composites with C was around 1 s, regardless of whether the MNs were the upper or lower layer and regardless of the grammage. This might be because the composites of A5 and A10 with C were all hydrophobic/hydrophilic structures, so when the hydrophobic layer (i.e., A5, A10) was the upper layer, it came into direct contact with the liquid, and the water fraction was transferred to the hydrophilic area from the hydrophobic area spontaneously under the action of the surface energy gradient. For the hydrophilic region, this wetting gradient effect was the cause of the phenomenon that MNs in the upper layer carried out the coffee filtration more quickly.

### 3.4. Purified Water Filtration Experiment Results and Analysis

The results of the experiments on the samples filtering pure water are shown through the figures below.

**Figure 10:**
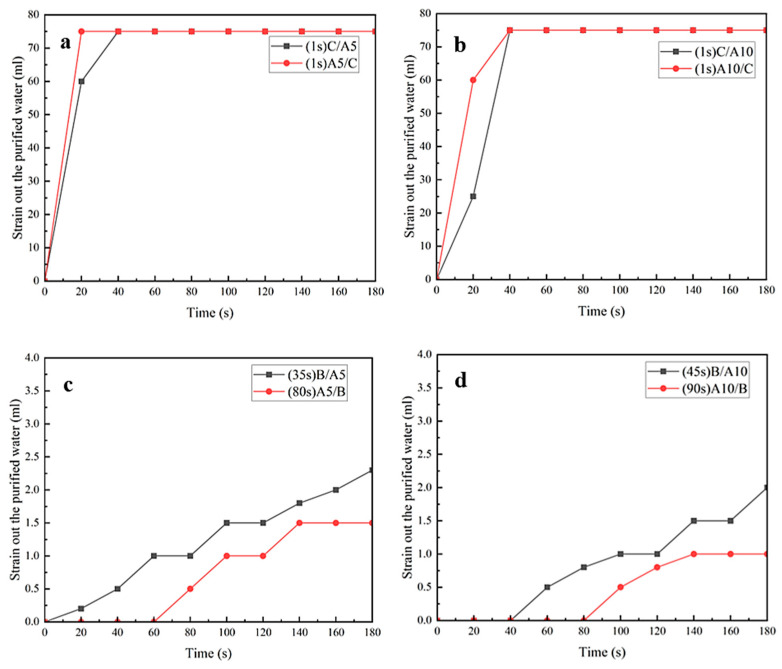
Comparison of the effect of D-LCCFNs on filtering purified water (**a**) A5/C and C/A5, (**b**) A10/C and C/A10, (**c**) A5/B and B/A5, (**d**) B/A10 and A10/B.

As can be seen from Figure 10a,b, the pure water filtration rates of both thicknesses of MNs were very fast when they were compounded with C, and all of them ended up filtering out about 75 mL of pure water. From Figure 10c,d, it can be seen that the pure water filtration rates were very slow and almost non-drip when the MNs were compounded with B. In addition, by comparing Figure 10a–d, it could be seen that the six laws of filtered coffee summarized above were met in the filtration of pure water in these samples.

The main difference between the samples of filtered water and filtered coffee prepared in this study was that the composites without hydrophilic material had a very low drip rate and a very small amount of filtered water when filtering pure water, and less than 3 mL of pure water was filtered at the end of the filtration.

**Figure 11:**
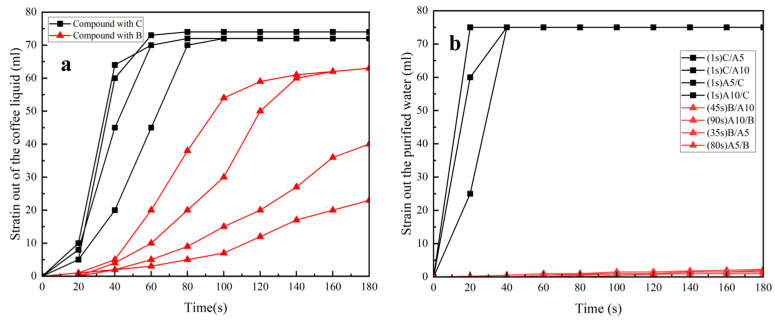
Filtration effect of the samples when filtering coffee and pure water, respectively. (**a**) Filtration effect of all samples when brewing coffee, (**b**) filtration effect of purified water brewed from all samples.

Figure 11a,b show the filtration effects of the samples when filtering coffee and pure water, respectively. The black square lines in both figures are the D-LCCFNs after compounding with a hydrophilic nonwoven (C), and the red triangle lines are the D-LCCFNs after compounding with a hydrophobic nonwoven (B). As can be seen from the figures, whether it was filtering coffee or pure water, the composites with C completed the filtration very quickly, and ended the filtration at 60~80 s, while the rate of the composites without C varied in the filtration of coffee, and the composites without C had an extremely slow drip rate in the filtration of pure water. However, when filtering coffee, it could be clearly seen that the amount of coffee filtration increased with the increase in time. 

This might be because the coffee contained a certain amount of oils and active molecules, which were extracted during filtration and came into contact with the nonwovens, where they adhered to the nonwoven and changed its surface roughness and pore structure and consequently changed the hydrophilicity of the nonwovens.

**Figure 12:**
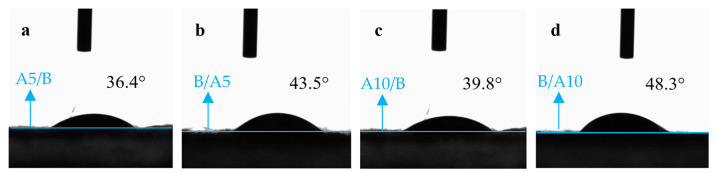
Test results of samples contact angle after coffee filtering, (**a**) A5/B, (**b**) B/A5, (**c**) A10/B and (**d**) B/A10.

To verify this conjecture, the A5/B, B/A5, A10/B and B/A10 specimens, which had been used to filter the coffee, were dried sufficiently and then tested again for contact angle, and the test results are shown in Figure 12.

Comparing the contact angle test results of D-LCCFNs before filtering coffee and after filtering coffee (Figure 3 and Figure 12), it can be seen that the contact angle of A5/B changed from 113.5° to 36.4°, the contact angle of B/A5 changed from 125.5° to 43.5°, the contact angle of A10/B decreased from 113.4° to 39.8° and the contact angle of B/A10 decreased from 119.4° to 48.3°; the contact angles of the four samples decreased by 70° to 82°, among which the contact angle that changed the most was B/A5, whose degree decreased by 82°. The contact angles of the samples after filtering the coffee were significantly lower than those before filtering the coffee, and the hydrophilicity of the sample increased significantly after filtering the coffee. 

### 3.5. FTIR Analysis of Samples and Coffee Powder

**Figure 13:**
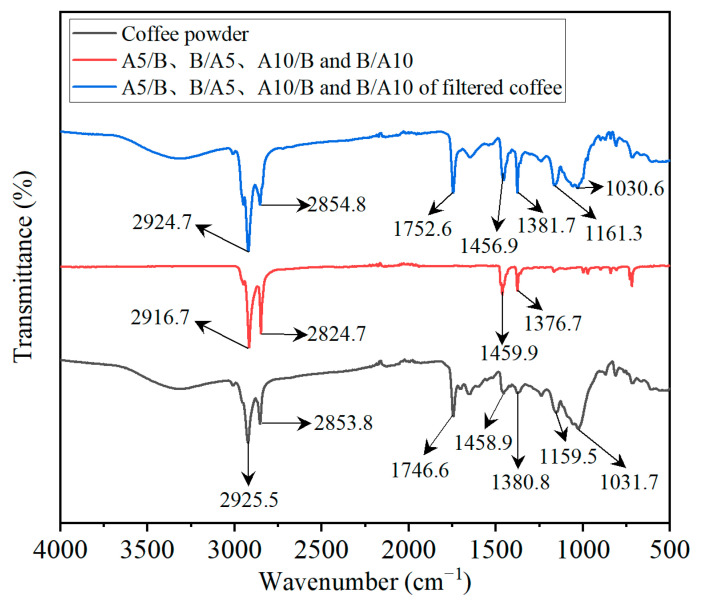
FTIR analysis results of coffee powder and D-LCCFNs before and after filtering coffee.

From the contact angle test results in 3.4, it was found that the contact angles of the D-LCCFNs samples without C (A5/B, B/A5, A10/B, B/A10) decreased after filtering the coffee. In order to investigate whether the change was due to the change of the physicochemical properties of these samples after coffee filtration, the coffee powder, the D-LCCFNs before filtering the coffee and the D-LCCFNs after filtering the coffee (after filtering the coffee, we dried the D-LCCFNs and removed the surface coffee powder) were subjected to FTIR spectroscopy tests, and the results of the tests are shown in Figure 13. 

As can be seen from Figure 13, the FTIR analysis results of D-LCCFNs (A5/B, B/A5, A10/B, B/A10) after coffee filtration were similar to the analysis test results of coffee powder, which was because the D-LCCFNs adsorbed a layer of coffee powder on their surface after coffee filtration. The raw materials of A5/B, B/A5, A10/B and B/A10 were all PP, and the comparison of these samples before and after coffee filtration showed that their main groups were not changed. Both of them showed -CH_2_-asymmetric stretching vibration in 2900 cm^−1^–2950 cm^−1^, -CH_2_- symmetric stretching vibration in 2850 cm^−1^–2860 cm^−1^, -CH_2_- symmetric stretching vibration near 1459 cm^−1^, -CH_2_- symmetric stretching vibration near 1459 cm^−1^, -CH_2_- bending vibration near 1459 cm^−1^ and -CH_3_ bending vibration near 1378 cm^−1^. This may indicate that when D-LCCFNs filter coffee, they mainly filter and adsorb coffee powder and extracted coffee flavor substances, while these samples and coffee did not chemically react and no valence bond was formed.

## 4. Conclusions

Based on market research results, this study used two areal densities of PP MNs, PP spunbonded nonwovens and viscose/ES chemically bonded nonwovens, as raw materials to prepare 8 types of D-LCCFNs for ear-hanging coffee filters. By characterizing the samples’ morphology and chemical structure as well as testing of the samples’ water contact angles, from the performances of coffee filtration and pure water filtration, the following conclusions were drawn:

(1) After compounding, each composite sample clearly showed a double-layer structure: one layer was ordinary spunbond nonwoven, and the other layer was an ultrafine fiber layer. The fibers in the ultrafine fiber layer were arranged in a disorderly manner, and the fibers were tangled and entangled. The PP spunbond nonwoven layer had flat nipping points, and the fibers in the viscose/ES nonwoven fabric had gelatin.
(2) For the D-LCCFNs, the effect of the surface density of the microfiber layer on the coffee filtration effect was more obvious, and the greater the surface density of the microfiber layer, the slower the coffee filtration; when PP MNs were compounded with PP spunbonded nonwovens, the rate of coffee filtration was slower in the upper layer of the MN, and the start of the drip filtration was later. When PP MNs were compounded with viscose/ES chemically bonded nonwovens, no matter whether the MNs were the upper layer or the lower layer and no matter how many grams the layer weighed, their start of drip filtration time was around the first second, and their coffee drip rate was faster and the amount of coffee liquid filtered out was larger.
(3) For the D-LCCFNs without hydrophilic materials, the drip rate was very slow and the amount of filtered water was very little when filtering pure water, but they could be used for coffee filtration, which might be due to the fact that coffee contained a certain amount of oils and activated molecules, and during filtration, the substances were extracted and came into contact with the nonwovens, which adhered to the nonwovens, altering the surface roughness and pore structure of nonwovens, and thus changing the hydrophilicity of the nonwovens.
(4) When coffee was filtered, D-LCCFNs mainly filtered and adsorbed coffee powder and extracted coffee flavor substances, and D-LCCFNs did not react chemically with coffee.

This study initially explored the coffee filtration law of double-layer structured nonwovens, but the detailed coffee filtration mechanism is not yet known. In the future, the coffee filtration mechanism of double-layer structured nonwovens may be studied thoroughly starting from the direction of molecules’ movement change in coffee during coffee filtration, and more suitable methods can be selected for the design and preparation of double-layer nonwoven coffee filtration fabrics when the mechanism is thoroughly investigated. 

## Data Availability

The original contributions presented in the study are included in the article, and further inquiries can be directed to the corresponding author.
